# TLFND: A Multimodal Fusion Model Based on Three-Level Feature Matching Distance for Fake News Detection

**DOI:** 10.3390/e25111533

**Published:** 2023-11-10

**Authors:** Junda Wang, Jeffrey Zheng, Shaowen Yao, Rui Wang, Hong Du

**Affiliations:** 1 Engineering Research Center of Cyberspace, Yunnan University, Kunming 650091, China; 2 School of Software, Yunnan University, Kunming 650091, China

**Keywords:** fake news detection, deep learning, matching distance, multimodal integrated detection

## Abstract

In the rapidly evolving information era, the dissemination of information has become swifter and more extensive. Fake news, in particular, spreads more rapidly and is produced at a lower cost compared to genuine news. While researchers have developed various methods for the automated detection of fake news, challenges such as the presence of multimodal information in news articles or insufficient multimodal data have hindered their detection efficacy. To address these challenges, we introduce a novel multimodal fusion model (TLFND) based on a three-level feature matching distance approach for fake news detection. TLFND comprises four core components: a two-level text feature extraction module, an image extraction and fusion module, a three-level feature matching score module, and a multimodal integrated recognition module. This model seamlessly combines two levels of text information (headline and body) and image data (multi-image fusion) within news articles. Notably, we introduce the Chebyshev distance metric for the first time to calculate matching scores among these three modalities. Additionally, we design an adaptive evolutionary algorithm for computing the loss functions of the four model components. Our comprehensive experiments on three real-world publicly available datasets validate the effectiveness of our proposed model, with remarkable improvements demonstrated across all four evaluation metrics for the PolitiFact, GossipCop, and Twitter datasets, resulting in an F1 score increase of 6.6%, 2.9%, and 2.3%, respectively.

## 1. Introduction

The popularization of the Internet has significantly increased the role of social platforms in people’s daily lives, serving as convenient channels for communication and information exchange [1]. Consequently, the number of social platform users has grown, leading to an exponential increase in the volume of information data. However, this growth has also given rise to the pervasive issue of fake news dissemination on these platforms, irrespective of its authenticity. This phenomenon is driven by the platforms’ pursuit of rapid development rather than a commitment to truth [2]. The spread of fake news poses a grave threat to information security and exerts a substantial influence on politics [3], the economy [4], and individuals’ well-being, resulting in immeasurable harm to society at large [5].

During the 2018 presidential elections in Brazil, false news regarding candidate Fernando Adula Bosonaro circulated [6]. These news articles claimed that Bosonaro’s opponent supported terrorism and abortion, leading to widespread misconceptions and prejudice against the opponent. In the same year, a fake news story about the Amazon rainforest fires spread rapidly on social media [7]. The fake news claimed that the Amazon rainforest fires were caused by arson by environmental groups, and this false information misinformed the public about rainforest conservation and sparked many false discussions and accusations. Additionally, in 2017, fake news falsely claimed that a bitcoin exchange had been hacked [8]. This resulted in a significant decline in the price of bitcoin, triggering panic among investors who hastily sold their bitcoins, thereby causing substantial volatility and losses within the bitcoin market.

As these problems have become more and more serious, research scholars have come up with ways to automatically detect fake news [9,10]. Initially, fake news detection primarily relied on textual content analysis [11,12,13,14]. However, as machine learning techniques advanced, support vector machines (SVM) were employed to identify fake news. It should be noted that SVMs alone may not fully capture the intricate relationships present in news texts [15]. Furthermore, the naive Bayesian classifier was also utilized as a baseline model, serving a similar purpose to SVM [16]. Subsequently, deep learning techniques gained prominence, with the aforementioned models becoming prevalent across various tasks. For instance, Goldani et al. [17] successfully applied a capsule network, originally used in computer vision, to detect fake news. In a similar vein, Raza et al. [18] integrated news content and contextual information using the Transformer architecture for detection. Notably, as the inclination to share news articles with images grew, researchers recognized the potential of incorporating visual elements in the detection process.

Scholars have embarked on investigating the detection of fake news in a multimodal manner. Giachanou et al. [19] employed neural networks to integrate text, visual, and semantic components. They utilized pre-trained GoogleNews-vectors-negative word vector models and the lexicon of affective reasoning (VADER) to process textual information, while also incorporating image tagging and LBP for the visual aspect. Similarly, Xue et al. [20] took into account the extrinsic and intrinsic characteristics of fake news and combines them with the overall consistency to identify the differences in their features, especially in the images where a branching network is designed to enhance their message. Additionally, Wu et al. [21] capitalized on people’s news consumption habits by comparing text and images. They designed a two-layer co-attention mechanism for image–text information, employed VGG to model the latent features of images, employed CNN to process the frequency domain information of images, and used co-attention to fuse the frequency and latent domain information. Finally, they combined these features with BERT for fake news detection. However, all of these multimodal approaches overlooked the fact that news headlines also constitute textual content and failed to account for the presence of multiple images within a single news article. Consequently, these limitations have resulted in unsatisfactory detection outcomes.

To address the following issues: (1) the limited utilization of multimodal information in unimodal detection, leading to unsatisfactory results; (2) the underutilization of multimodal information in recent multimodal detection approaches, such as neglecting headlines and multiple images; and (3) the simplistic fusion and detection of multimodal information without comprehensive consideration of matching distances between features, we propose an innovative model called the Three-Level Feature-Based Matching Distance Multimodal Fusion Model (TLFND). TLFND is based on RoBERTa [22] for extracting two levels of text features (title and body) and VGG-19 for obtaining image features. We incorporate bidirectional long- and short-term memory (BILSTM) in image fusion to enhance the comprehensiveness of image features. Furthermore, we calculate the Chebyshev distance metric between the three features to determine the veracity of news, bringing them into a unified dimensional space. The model is optimized using a hinge loss function with a predefined threshold. Specifically, for true news with a label value of 0, we aim to maximize the distances, while for true news with a label value of 1, we strive to minimize the three-way distance below the threshold.

Based on the above contributions, the following summarization is presented:

- Taking into account the strengths and weaknesses of previous research, we have developed an innovative model called TLFND (Three-Level Feature Similarity Distance-based Multimodal Fusion Model) for detecting fake news. This model effectively harnesses the power of news headlines, textual content, and multiple images to accurately identify fake news.
- We propose an auxiliary task that uses the Chebyshev distance metric function as a distance metric between multiple modalities; calculates the distance to the center of mass using headlines, text, and fused images; optimizes the model using HingeEmbeddingLoss with set thresholds; and finally, designs an adaptive evolutionary algorithm to calculate the loss functions of the four components in order to detect false news more accurately.
- We conducted a comprehensive evaluation of the TLFND model using three widely used real-world datasets (PolitiFact, GossipCop, and Twitter). Through multiple sets of experiments and the incorporation of various evaluation metrics, our findings consistently demonstrated the superior performance of TLFND when compared to the current state-of-the-art multimodal fake news detection models.

The following sections of this paper are organized as follows: In Section 2, we provide a comprehensive review of previous research efforts in the field of fake news detection. Section 3 focuses on the symbolic representations used in this paper and discusses the structural framework of the TLFND model. In Section 4, we delve into the various components of the TLFND model, providing detailed explanations of the technical aspects involved. Section 5 presents the experimental setup, including the dataset, experimental parameters, baseline, and comparison results. Finally, Section 6 concludes our study with a comprehensive summary of our findings.

## 2. Related Work

In this section, we provide a comprehensive overview of the existing methods proposed for the automatic detection of fake news. We categorize these methods into two groups: unimodal detection methods and multimodal detection methods. For each method, we examine the key techniques employed and the progress made in the field. Additionally, we emphasize the strengths and limitations of each approach.

### 2.1. Single-Mode Detection Method

The unimodal approach to fake news detection focuses on analyzing a single modality, such as text, image, or video, by identifying the features within that modality. For instance, Ma et al. [23] employed deep learning techniques, including RNN, LSTM, and GRU, to convert text into a vector representation and feed it into a classifier for results. However, they overlooked other important textual information such as headlines and comments. In a novel approach, Yu et al. [24] developed a perceptual framework that combines the news environment and domain history. By considering the contextual factors, they designed a perceptual recognition module and achieved significant improvements by incorporating domain fusion. Kausar et al. [25] utilized n-grams with TF-IDF for word embedding to extract content features and trained LSTM and BERT models to process news contextual features. They then employed a feedforward neural network for classification. However, their approach did not consider the comprehensive utilization of multiple textual features. Addressing the issue of biased information, Liao et al. [26] studied topic tags and authors’ historical postings. They employed representation learning and multi-task learning, combined with a dynamic weighting strategy to reduce risks, and achieved a promising detection performance. Bazmi et al. [27] recognized the credibility disparities between users and news based on differences in news topic viewpoints. By integrating the viewpoint, source bias, and user bias, they constructed three corresponding components and applied joint coding to detect biases through simulated interactions. However, their method did not fully consider the dynamic nature of news dissemination. To address this limitation, Song et al. [28] proposed a dynamic graph neural network that incorporates temporal information from news dissemination graphs. By generating dynamic representations using a perception module, they improved false news detection by capturing the temporal dynamics. In another approach, Kumar et al. [29] developed a hybrid model that optimized feature selection based on usefulness. They initially extracted features using TF-IDF for weighting and then employed the MGO algorithm to select the most salient features. Finally, they combined the selected features for detection. However, their approach did not fully exploit the multimodal nature of news, which often contains multiple types of information. A common drawback in the aforementioned studies is that they only extract features from a single modality for prediction, while contemporary news encompasses multiple modalities. This limitation hinders the comprehensive utilization of news information for effective detection.

### 2.2. Multimodal Detection Methods

To overcome the limitations of single modality approaches, researchers have increasingly turned to multimodal techniques. Advanced feature extractors such as BERT and Transformer for text and VGG and ResNet for visuals have emerged to facilitate convenient multimodal fusion in detection tasks. Song et al. [30] utilized multimodal adversarial multitask learning to capture and homogenize news article feature distributions from different domains. They employed the transformer KAT to enhance the selective embedding of entities from external knowledge graphs, resulting in improved performance. The attention mechanism has also been explored for news tasks. Guo et al. [31] employed an attention mechanism neural network to enhance modal fusion. They designed a structured framework to protect middle-layer information, preventing information loss during fusion and thereby enhancing detection. In a different approach, Li et al. [32] proposed semantic enhancement for fusion. They combined textual, visual, and semantic information by leveraging snapshot techniques, which involved integrating these different modalities. They designed an adaptive network to classify special and shared features, reducing errors in the fusion process. Wang et al. [33] not only fused different modalities but also considered various image features. They incorporated attention mechanisms to jointly combine correlations and dependencies between the features, enhancing fusion in the process. Metric calculations have also been introduced. Chen et al. [34] proposed aligning modal features by mapping features from different modalities into an embedding space. They measured the distribution between single modalities using KL scatter. However, these models often require complex parameter settings. To address this, Singh et al. [35] designed a stacked framework. For text processing, they employed BERT and ELECTRA, while for images, they utilized the efficient NasNet Mobile. This framework not only achieved excellent detection results but also reduced the overall parameters by about 20%, image parameters by 2%, and text parameters by 60%.

## 3. Problem Statement

In this section, we discuss the symbolic representations utilized in this paper and describe the application process of the proposed TLFND model. In a news article, various components such as the title, content, image, and label (indicating true or false news) are present. We represent the collection of news articles as $N_{i}=\left\{\left(T_{i},C_{i},S_{i}\left\{V_{j},V_{j+1},\cdots \right\},B_{i}\right)\right\}$, the title as $T_{i}$, the content as $C_{i}$, the collection of images as $S_{i}$, a single image in a news article as $V_{i}$, and the label as $B_{i}$. A false news article is denoted by $B_{i}=0$, while a true news article is represented by $B_{i}=1$.

To begin, we extract $T_{i}$ features $T_{i}^{r}$ and $C_{i}$ features $C_{i}^{r}$ using the RoBERTa model (described in Section 4.1). Subsequently, we extract individual $V_{i}$ features $V_{i}^{r}$ using the pre-trained VGG-19 model (described in Section 4.2). Afterward, we fuse the multiple $V_{i}^{r}$ features into $S_{i}^{r}$ in a BILSTM model operating in two opposite directions (described in Section 4.2). Next, we concatenate $T_{i}^{r}$, $C_{i}^{r}$, and $S_{i}^{r}$ to create a multimodal feature vector denoted as $E_{i}^{r}$. Finally, we input $E_{i}^{r}$ and $B_{i}$ into the matching distance calculation module (described in Section 4.3).

## 4. Proposed Method

**Figure 1:**
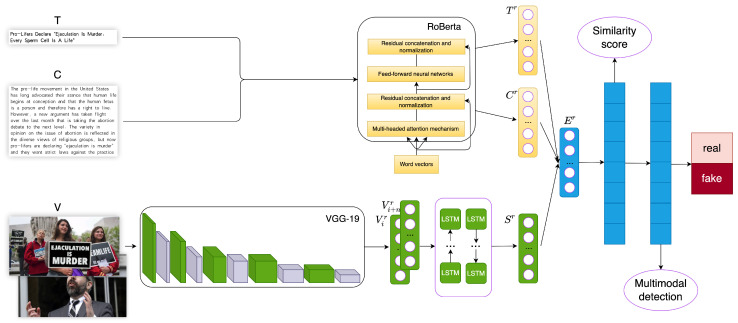
Structure diagram of the proposed multimodal model TLFND for false news detection. T represents the news headline, C represents the news content, and V represents the images in the news.

In response to the existing problems of previous generations, we propose a new TLFND model that combines the RoBERTa model and the VGG-19 model to address the limitations of previous studies. We recognize that a news article contains not only the body text but also the headline, and there may exist inconsistencies among the headline, the body text, and the accompanying image. However, many researchers have overlooked this issue and focused solely on the matching distance between the body text and the image. Additionally, news articles often include multiple images, yet researchers often only consider the first image when constructing their datasets. To address these challenges, we extract text features (headline and body text) and fuse them with multi-image features. Finally, we utilize the Chebyshev distance metric to calculate the matching distance of the fused vector features, enabling the detection of false news. The overall structure of our proposed TLFND model is illustrated in Figure 1.

The model consists of the following parts:

1. Two-level text feature extraction module.
2. Image extraction and fusion module.
3. Three-level feature matching distance module.
4. Multimodal integration recognition module.

### 4.1. Two-Level Text Feature Extraction Module

The traditional technique Word2Vec [36] represenst each word as an independent vector, without considering the intricate relationships between words and their surrounding contexts. Notably, the BERT model [37] has demonstrated promising results across various tasks. To achieve optimal detection performance, we leverage an enhanced variant of the BERT model, namely RoBERTa. The RoBERTa model surpasses its predecessor by utilizing a larger dataset and longer training time, enabling it to learn deeper linguistic representations and richer semantic information [38]. However, RoBERTa also performs exceptionally well in sentiment analysis, thanks to its deeper language representations [39]. Furthermore, some researchers have suggested that RoBERTa exhibits advantages over other state-of-the-art Transformer architectures in tasks related to text classification and detection [40,41]. Subsequently, we define two fully connected layers, each comprising a linear layer and a ReLU activation function. Dropout layers are employed to randomly discard output from the RoBERTa model, thereby mitigating the risk of overfitting.

### 4.2. Image Extraction and Fusion Module

VGG [42] is a convolutional neural network architecture developed by Oxford University, while VGG-19 is a specific variant of VGG, characterized by a simple structure that facilitates a better understanding of its working implications and parameters. It also exhibits a strong generalization capability, which makes it a popular base model and contributes to its improved performance in various tasks. The VGG-19 model comprises multiple convolutional layers with a 3×3 kernel size and pooling operations with a 2×2 size stacked together. This design enhances the expressiveness of the model and its ability to extract features. In our approach, we avoid converting the image information into textual representations, as this may lead to information loss. Instead, we input a single image into the VGG-19 model, remove its classifier layer, and then pass the extracted features through a fully connected layer to map them to a lower-dimensional representation. Subsequently, the hidden states are combined in two opposite directions using BILSTM and mapped to the final $S^{r}$.

### 4.3. Three-Level Feature Matching Distance Module

We apply fully connected layers to map $T^{r}$, $C^{r}$, and $S^{r}$ to a shared space. These mapped representations are then stitched together to form a multimodal feature vector $E^{r}$. $E^{r}$ is a three-dimensional matrix with a shape of p×q×n, where *p* represents the number of samples, *q* represents the number of text and image features, and *n* represents the dimension of the multimodal space (set to 128). To calculate the center of mass *M* in dimension 1, we utilize Equation (1), where *L* denotes the number of samples and $x_{i}$ denotes the feature vector of the ith sample. Following this, the center of mass *M* is transformed into a matrix of the same size as $E^{r}$, as shown in Equation (2). We then employ the Chebyshev distance to compute the distance between $E^{r}$ and $M_{reshape}$, as illustrated in Equation (3). Here, PT denotes the Chebyshev distance function, *i* represents the first few samples, and *L* represents the number of samples. Finally, we compute the average HingeEmbeddingLoss of all sample pairs in dist_mat using PyTorch’s built-in loss function, HingeEmbeddingLoss, as shown in Equation (3). We set a predefined threshold value to minimize the distance between matched sample pairs (label value = 1) and maximize the distance between mismatched sample pairs (label value = 0). Therefore, the objective of using HingeEmbeddingLoss is to optimize the model parameters in such a way that the distance between positive samples is minimized, while the distance between negative samples is maximized. This optimization aims to enhance the performance and discriminative power of the model, as depicted in Equation (4).

(1) $$M=\frac{1}{H}\sum_{i=1}^{H}x_{i}$$

(2) $$M_{reshape}=M_{p\times q\times n}$$

(3) $$dist\_mat=\frac{1}{H}\sum_{i=1}^{H}PT{\left(E^{r},\;M_{reshape}\right)}_{i}$$

(4) $$Hing\_ls=\frac{1}{L}\sum_{i=1}^{L}HINGE\_LOSS\left(y_{i},{dist\_mat}_{i}\right)$$

### 4.4. Multimodal Integration Recognition Module

Combining the aforementioned three modules, we propose the primary objective of this paper, namely, a multimodal integrated recognizer. Firstly, we pass the outcomes of the three modules mentioned above into the multimodal integrated recognition module. Three prediction results ($N_{TC}$, $N_{S}$, $N_{E}$) are defined to compute the final loss function. The text feature vector and title feature vector are concatenated and subjected to linear transformation through the fully connected layer, yielding $N_{TC}$, as depicted in Equation (5). Similarly, the image features undergo linear transformation to obtain $N_{S}$, as shown in Equation (6). Furthermore, the fused multimodal features are linearly transformed to obtain $N_{E}$, as illustrated in Equation (7). The corresponding weight matrix and bias vector are employed for the calculations.

(5) $$N_{TC}=\left[T_{r},C_{r}\right]*W_{TC}+B_{TC}$$

(6) $$N_{S}=S_{r}*W_{S}+B_{S}$$

(7) $$N_{E}=E_{r}*W_{E}+B_{E}$$

### 4.5. Adaptive Evolutionary Loss

In the TLFND fake news detection model, the design of the overall loss function is crucial, and we calculate a total of four partial loss functions and design am adaptive evolutionary algorithm, as shown in Algorithm 1. The weight-adjusted values of the relative differences of the loss values are generated to weight the different loss values according to their relative importance to better balance the contributions of different losses. Finally, the weighted summation is performed using the weight-adjusted loss values and the corresponding weights. Combined with the above character representation, we set the following four loss values: ${LN}_{TC}$: cross-entropy loss calculation for $N_{TC}$; ${LN}_{S}$: cross-entropy loss calculation for $N_{S}$; ${LN}_{E}$: cross-entropy loss calculation for $N_{E}$; ${LN}_{M}$: matching distance loss calculation (Section 4.3). For ${LN}_{TC}$, ${LN}_{S}$, and ${LN}_{E}$ the calculation is shown in Equation (8).

(8) $$LN=-\sum \left(y*\ln\left(p\right)\right)$$

**Algorithm 1** Loss adaptive evolutionary algorithm

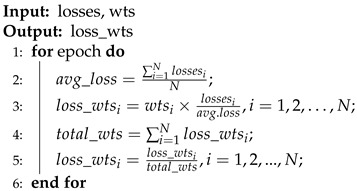



## 5. Experiments and Results

In this section, we provide a comprehensive overview of the experimental setup, which encompasses the dataset description, parameter settings, and a comparison of baselines along with the evaluation metrics.

### 5.1. Datasets

We validated the performance of the TLFND model using a fake news detection database called FakenewsNet DataSet (consisting of two datasets, PolitiFact and GossipCop) [43] in conjunction with a dataset, Twitter, applied to the Scalable Multimedia task of MediaEval 2016 [44].

1. PolitiFact dataset. The PolitiFact dataset focuses on politically relevant news and incorporates fact-checking information gathered from the PolitiFact website (www.politifact.com accessed on 15 June 2023). It includes news articles, fact checks, and associated comments from social media platforms. Fact checkers have rated each news article as true, partially true, or false. Each news article within this dataset contains various details, such as text content, news image address, posting time, author information, and social media comments.
2. GossipCop dataset. The GossipCop dataset, on the other hand, revolves around entertainment and celebrity news. It consists of entertainment news articles and related comments obtained from the GossipCop website (www.gossipcop.com accessed on 15 June 2023). Similar to the PolitiFact dataset, each news article within the GossipCop dataset includes information like text content, news image address, posting time, author information, and associated social media comments. The news items in this dataset have been rated as either true or false.
3. Twitter dataset. The Twitter dataset is an English-language dataset containing images and text released for the MediaEval multimedia task, and in order to validate the model performance of TLFND in multiple scenarios, we will use tweets containing text and image content, filtering out other tweets (e.g., with video, etc.).

To adapt the TLFND model, we extracted a subset of data from the FakenewsNet DataSet, which is a database specifically designed for fake news detection. We focused on collecting news headlines, content, and images while excluding publication time, author information, and user comments, as these data were not relevant to our model. Additionally, we removed news articles that did not contain any images. In total, we collected 853 data samples from the PolitiFact dataset, consisting of 529 real news items and 326 fake news items. Similarly, we collected 15,613 data samples from the GossipCop dataset, including 12,119 real news items and 3494 fake news items. Finally, 11,261 data—4425 real data and 6836 fake data—were collected in the Twitter dataset. All three datasets were divided into training and test sets using a 5:1 ratio, as presented in Table 1.

### 5.2. Evaluation Metrics

In this section, evaluation metrics for assessing the fake news detection model TLFND, including accuracy, recall, precision, and F1 score, are presented in Equations (9), (10), (11), and (12), respectively.

(9) $$\mathrm{Accuracy}=\frac{TP+TN}{TP+TN+FP+FN}$$

(10) $$\mathrm{Recall}=\frac{TP}{TP+FN}$$

(11) $$\mathrm{Precision}=\frac{TP+TN}{TP+FP}$$

(12) $$F1\;\mathrm{Score}=\frac{2\times (\mathrm{Precision}\times \mathrm{Recall})}{\mathrm{Precision}+\mathrm{Recal}}$$

### 5.3. Model Parameters

We conducted our experiments on an NVIDIA RTX 4090 GPU with 24 GB of RAM, utilizing CUDA version 11.0 for implementation. In our setup, we defined the maximum input length for news headlines as 100 and for news content as 510. We extracted headline and content dimensions of 128 and 512, respectively. For the input images, we resized them to 224×224 pixels. Subsequently, the images were processed through two fully connected layers of VGG-19, with the first fully connected layer having a dimension of 1024 and the second fully connected layer outputting a dimension of 256. To prevent overfitting and enhance model convergence, we employed the AdamW optimizer with a learning rate of $1\times 10^{-5}$. Furthermore, to improve the generalization ability of the model, we applied a dropout rate of 0.4. In both datasets, we use an approximate ratio of 5:1 to divide the dataset for training and testing. During the training phase, each batch consisted of sixteen samples (train_batch_size), while during the evaluation phase, each batch contained one sample (eval_batch_size). We conducted a total of 50 training sessions (epochs), with the specific parameter settings detailed in Table 2.

### 5.4. Baselines

We conducted experiments using the FakenewsNet database (PolitiFact and GossipCop) and the Twitter dataset. We compared the results of two types of models: single-peak detection models and multi-peak detection models.

#### 5.4.1. Single-Mode Detection

SVM [15]: The SVM classifier classifies news articles by identifying an optimal hyperplane within the feature space, which includes relevant features such as word frequency and word vectors, social media features like the number of retweets and likes, and structural features such as headline length and paragraph structure. In our dataset, we utilize text features to train the SVM model and achieve a good fit.

CNN [45]: We employed a state-of-the-art shallow convolutional neural network (CNN) specifically designed for this type of task, which previously secured first place in the 9th International Competition on Authorship [46]. This network processes the samples from the dataset, constructs a dictionary, and customizes a preprocessing function for generating n-grams. Finally, the output is fed into a fully connected global pooling layer.

VGG-19 [42]: VGG-19 is a variant of the VGG model that can effectively extract high-level features of images and capture details and semantic information in images by stacking multiple convolutional and pooling layers.

#### 5.4.2. Multimodal Detection

SAFE [47]: The model is a representative work for recognizing the similarity of news images and texts. The image2text model is used to transform the images into corresponding headlines, which are then mapped to the same vector space as the text, and an improved cosine similarity is proposed for recognition.

SpotFake+ [48]: This author summarizes the drawbacks of last year’s study by introducing migration learning into the model and training it for recognizing semantics and exploring contextual relationships, processing images and text using VGG and XLNET, and feeding the training results into a fully-connected layer for classification.

DEFD [49]: The model utilizes the integration of deep learning and attention mechanisms to obtain text features from pre-trained XLNet and image features from pre-trained VGG-19. A hybrid feature loss function is designed to reduce the classification error, and finally, the final prediction results are output using a weighting mechanism.

BCMF [50]: The method is presented in a journal with a 5-year average factor of 7.4 and uses a contextual pre-trained visual model, namely DEIT, to process text with the help of BERT and proposes a novel mechanism, i.e., text to image and image to text, in a bidirectional loop. Finally, the detection is performed by FFN input to the Softmax function.

### 5.5. Experimental Results and Analysis

In order to be able to verify the performance of the TLFND model in all aspects, we tested it in three different groups of experiments.

#### 5.5.1. Comparison Experiments

We use the PolitiFact, GossipCop, and Twitter datasets as experimental datasets and the evaluation metrics in Section 5.2 as the experimental metrics and compare the two types of models in Section 5.4. The results are shown in Table 3, Table 4 and Table 5.

**Figure 2:**
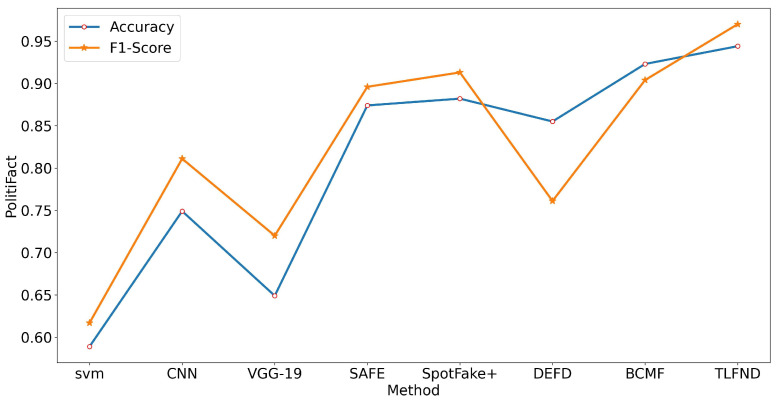
Line graph representation of the accuracy and F1 score metrics in the PolitiFact dataset baseline approach versus the TLFND model.

**Figure 3:**
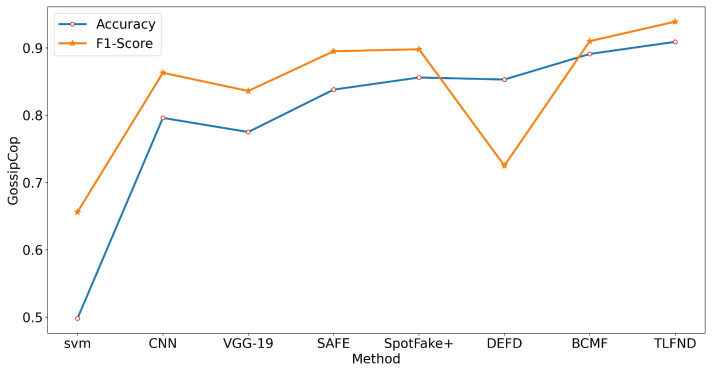
Line graph representation of the accuracy and F1 score metrics in the GossipCop dataset baseline approach versus the TLFND model.

**Figure 4:**
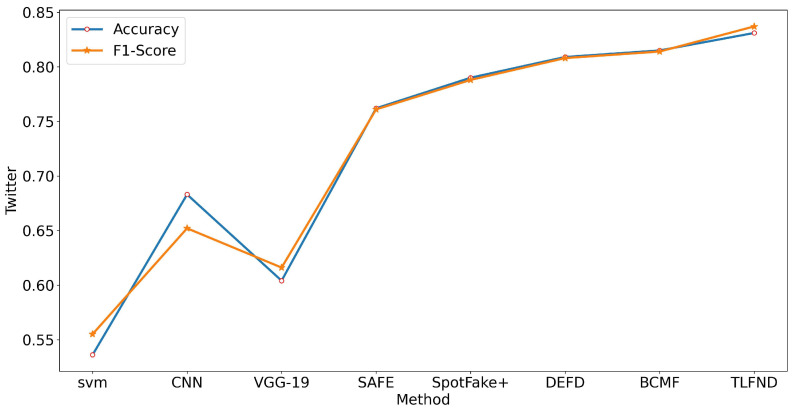
Line graph representation of the accuracy and F1 score metrics in the Twitter dataset baseline approach versus the TLFND model.

According to the experimental results, TLFND shows a superior performance to other unimodal and multimodal methods on all four evaluation metrics for all three datasets. Specifically, TLFND achieved an accuracy of 94.4% and an F1 score of 97% on the PolitiFact dataset, representing a 2.1% improvement in accuracy and a 5.7% improvement in F1 score compared to the current state-of-the-art methods. On the GossipCop dataset, TLFND achieved an accuracy of 90.9% and an F1 score of 93.9%, indicating a 1.8% improvement in accuracy and a 2.9% improvement in F1 score compared to the current state-of-the-art method. On the Twitter dataset, TLFND achieved an accuracy of 83.3% and an F1 score of 83.7%, indicating a 1.6% improvement in accuracy and a 2.3% improvement in F1 score compared to the current state-of-the-art method. To provide a visual representation of the differences in evaluation metrics, we have included line graphs depicting the experimental results on both datasets (refer to Figure 2, Figure 3 and Figure 4).

Multimodal methods outperform unimodal methods such as the SAFE model and the CNN model on both datasets. The SAFE model represents a significant advancement in utilizing both news headlines and images. However, it does not directly compare images; instead, it transforms images into text. This transformation can lead to a loss of thematic meaning and may be one of the reasons for the SAFE model’s relatively poor performance in multimodal tasks. In contrast, the CNN model is the most effective model among the unimodal methods, but its performance is still inferior to that of the MAVE model, which further confirms that the performance and accuracy of the model can be improved by fusing information from different modalities.

Based on the above, the TLFND model performs best, and we guess that it is mainly due to the following reasons:

1. TLFND extracts text features using the RoBERTa model, which is trained with a higher number of iterations compared to the BERT model as well as a dynamic masking strategy, thus improving the model’s ability to understand and generalize the context. The text features are not only the body content, but also the title, which is very important for news, and the keywords, event descriptions, and other information in the title also influence the body content. We used the VGG-19 model to extract image features. Usually, a news article will have multiple images, and the multimodal approach does not introduce multiple images; thus, we use BILSTM to fuse multiple image features for better understanding of news information.
2. We designed a multimodal matching distance module, which will extract the title features, body features, and fused image features and stitch them into a multimodal feature vector. We then use the Chebyshev distance to calculate the distance of the three features from the center of mass and set a threshold such that, if the sample pair label is true news, then distance between the sample pairs is as small as possible compared to the threshold, but if the sample pair label is false news, then the distance between the sample pairs is as large as possible compared to the threshold. This feature should improve the performance and discriminative ability of the model.
3. The loss function plays a crucial role in multimodal false news detection. It is used to measure the difference between the model prediction results and the true label. Designing a loss function suitable for the model can enable the model to learn more accurate and reliable prediction results. A loss function is designed in our proposed TLFND model, and a total of four parts of the loss function are calculated: F1, the feature vector of linear transformation of the text feature vector and title feature vector after stitching through the fully connected layer using cross-entropy; F2, the feature vector of linear transformation of the image features using cross-entropy; F3, the feature vector of fused multimodal features using cross-entropy; F4, the matching distance loss calculated using the HingeEmbeddingLoss loss function. Moreover, a dynamic weighting algorithm is designed to weight different loss values according to their relative importance to better balance the contributions of different losses. Finally, the weight-adjusted loss values are weighted and summed with the corresponding weights to serve as the combined loss values.

#### 5.5.2. Ablation Study

To assess the importance of each component to the model and its contribution to the overall performance and to help understand how the model works and the key factors, we conducted an ablation study.

The TLFND variants are as follows: TLFND-T: removed the visual information, multimodal information, and matching distance and used only text information. TLFND-C: removed the text information, multimodal information, and matching distance and used only visual information. TLFND-E: removed the matching distance and used text information, image information, and multimodal fusion. The experimental results are shown in Table 6.

From the results shown in Table 6, we have the following findings:

1. TLFND-C performs less well, which indicates that single text features are not equally important as single image features, and text detection is better in both datasets.
2. TLFND-E performs better than both TLFND-T and TLFND-C, which proves that fusing modalities is better than single modalities, and this confirms the research significance of multimodal news detection.
3. Ultimately, it is shown that the TLFND model is the best performer in terms of each metric, and each module has its own unique role that complements each other.

In summary, each module in the TLFND model has its own importance, and each module is indispensable to the TLFND model and has its own role in false news detection. The TLFND model that integrates the four modules to form the TLFND model performs well in false news detection.

#### 5.5.3. Matching Distance Module Analysis

In the TLFND model, we utilize the Chebyshev distance metric to calculate the matching distance between $T^{r}$, $C^{r}$, and $S^{r}$. To demonstrate the effectiveness of the Chebyshev distance metric in false news detection, we propose comparing it with different distance metrics. Specifically, we apply the following two alternative distance metrics to the TLFND model: TLFND-COS, which replaces the Chebyshev distance metric with cosine similarity, and TLFND-MD, which replaces the Chebyshev distance metric with Manhattan distance. The experimental results of this comparison are presented in Table 7.

From the results in Table 7, we can learn that using the Chebyshev matching measure in the similarity distance module performs better than the cosine similarity distance measure and the Manhattan distance measure. This may be due to the following reasons:

1. The Chebyshev distance metric considers the maximum absolute difference between vectors, i.e., the maximum difference is taken in each dimension, which can better capture the overall difference between vectors. In contrast, the Manhattan distance only considers the cumulative difference between vectors, while the cosine similarity metric only considers the angle between vectors.
2. The Chebyshev distance metric is able to capture the difference between modes more sensitively in the multimodal matching distance, while the Manhattan distance and cosine similarity metrics may be affected by the feature scale or distribution.

#### 5.5.4. Convergence Analysis

**Figure 5:**
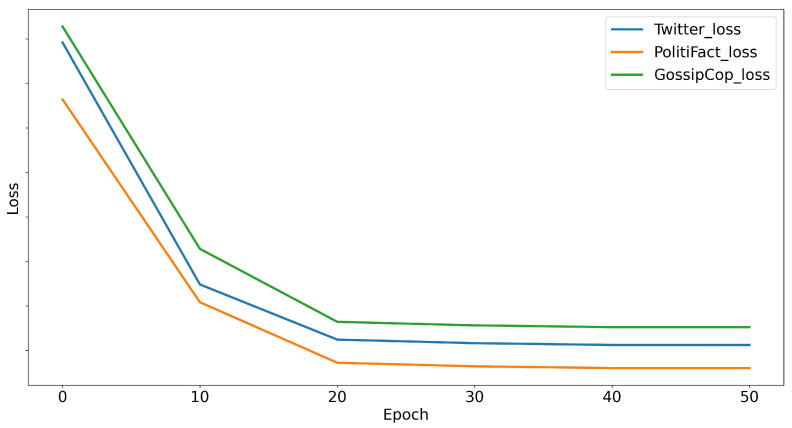
Mean loss curves for three different datasets.

To explore and analyze whether the TLFND model faces overfitting during training, we generated average loss iteration curves of the TLFND across three distinct datasets, as illustrated in Figure 5. From Figure 5, it is evident that the three losses progressively reduce at the outset and gradually stabilize later, signifying that the model achieves a certain equilibrium. To address the issue of overfitting, we first introduced the Dropout function within both the text and image processing functions. This function facilitates the random dropping of neurons during model training, thereby reducing the model’s susceptibility to overfitting on the training data. Subsequently, we incorporated L2 regularization via the AdamW optimizer, aiding in controlling the model’s complexity. Lastly, we divided the loss function into four parts and designed an adaptive evolutionary algorithm. This algorithm not only optimizes the model but also manages overfitting issues.

## 6. Conclusions

To enhance the performance of multimodal methods in fake news detection tasks, we propose a groundbreaking model called TLFND. This model is based on the RoBERTa and VGG-19 models, combining their strengths to create a powerful fake news detection system. The TLFND model comprises four components: a two-level text feature extraction module, an extracted and fused image module, a three-level feature matching distance module, and a multimodal integrated recognition module. Compared to existing multimodal models, the TLFND model takes a significant step forward by considering two types of text: headlines and body text. It effectively fuses these text sources with multiple news images using BILSTM, resulting in a comprehensive understanding of the news content. Additionally, we introduce the Chebyshev distance metric for the first time, enabling accurate calculation of the matching distance between the three distinct features. To optimize the model’s performance, we employ an adaptive evolutionary algorithm. This algorithm computes the loss values of the four components, facilitating parameter optimization and learning through feedback signals. The TLFND model evolves and improves iteratively, delivering superior performance in fake news detection. We evaluated the TLFND model on three real-world public datasets, and the experimental results demonstrate its superiority. In all four metrics, the TLFND model outperforms other approaches, securing the top position. This achievement showcases the practical application of the TLFND model in the crucial task of binary false news detection, effectively countering the spread and impact of false information. Moreover, the TLFND model serves as a valuable reference for researchers in the field and holds potential for broader applications in various domains.

Looking ahead, our future research efforts will focus on adapting the TLFND model to different domains and conducting extensive testing and refinement to enhance its effectiveness and technical details. By continuously improving the model, we aim to make meaningful contributions to society’s fight against fake news.

## Figures and Tables

**Table 1:** Statistics and segmentation of PolitiFact, GossipCop, and Twitter datasets.

Dataset	Train/Test	Real/Fake
Politifact	710/143	527/326
Gossipcop	13,011/2602	12,119/3494
Twitter	9384/1877	4425/6836

**Table 2:** Model parameter settings.

Parameters	Size
Epochs	50
Train_batch_size	16
Eval_batch_size	1
Optimizer	AdamW
Learning rate	$1\times 10^{-5}$
Dropout rate	0.4

**Table 3:** Results of comparison experiments using the PolitiFact dataset (bold represents the best results).

Method	Accuracy	Precision	Recall	F1 Score
SVM	0.589	0.467	0.911	0.617
CNN	0.749	0.786	0.837	0.811
VGG-19	0.649	0.668	0.787	0.720
SAFE	0.874	0.889	0.903	0.896
SpotFake+	0.882	0.903	0.925	0.913
DEFD	0.855	0.705	0.827	0.761
BCMF	0.923	0.904	0.904	0.904
TLFND	0.944	0.974	0.966	0.970

**Table 4:** Results of comparison experiments using the GossipCop dataset (bold represents the best results).

Method	Accuracy	Precision	Recall	F1-Score
SVM	0.498	0.523	0.883	0.656
CNN	0.796	0.831	0.897	0.863
VGG-19	0.775	0.793	0.884	0.836
SAFE	0.838	0.857	0.927	0.895
SpotFake+	0.856	0.879	0.918	0.898
DEFD	0.853	0.912	0.601	0.725
BCMF	0.891	0.902	0.919	0.910
TLFND	0.909	0.932	0.947	0.939

**Table 5:** Results of comparison experiments using the Twitter dataset (bold represents the best results).

Method	Accuracy	Precision	Recall	F1-Score
SVM	0.536	0.571	0.541	0.555
CNN	0.683	0.655	0.651	0.652
VGG-19	0.604	0.639	0.596	0.616
SAFE	0.762	0.763	0.767	0.761
SpotFake+	0.790	0.789	0.748	0.788
DEFD	0.809	0.825	0.793	0.808
BCMF	0.815	0.813	0.816	0.814
TLFND	0.831	0.852	0.824	0.837

**Table 6:** Ablation study on PolitiFact, GossipCop, and Twitter datasets for the TLFND model design (bold represents the best results).

Dataset	Method	Accuracy	F1 Score
Politifact	TLFND-T	0.896	0.893
	TLFND-C	0.828	0.831
	TLFND-E	0.912	0.899
	TLFND	0.944	0.970
GossipCop	TLFND-T	0.864	0.901
	TLFND-C	0.683	0.785
	TLFND-E	0.869	0.910
	TLFND	0.909	0.939
Twitter	TLFND-T	0.788	0.801
	TLFND-C	0.721	0.739
	TLFND-E	0.810	0.808
	TLFND	0.831	0.837

**Table 7:** Analysis of the matching distance module in the TLFND model on the GossipCop, PolitiFact, and Twitter datasets (bold represents the best results).

Dataset	Method	Accuracy	F1 Score
Politifact	TLFND-COS	0.919	0.935
	TLFND-MD	0.928	0.941
	TLFND	0.944	0.970
GossipCop	TLFND-COS	0.863	0.906
	TLFND-MD	0.875	0.913
	TLFND	0.909	0.939
Twitter	TLFND-COS	0.803	0.811
	TLFND-MD	0.819	0.815
	TLFND	0.831	0.837

## Data Availability

Data are contained within the article.
